# Bufalin-Loaded PEGylated Liposomes: Antitumor Efficacy, Acute Toxicity, and Tissue Distribution

**DOI:** 10.1186/s11671-019-3057-0

**Published:** 2019-07-05

**Authors:** Jiani Yuan, Cheng Zeng, Wei Cao, Xuanxuan Zhou, Yang Pan, Yanhua Xie, Yifang Zhang, Qian Yang, Siwang Wang

**Affiliations:** 1 Department of Chinese Materia Medica and Natural Medicines, School of Pharmacy, Air Force Medical University, Xi’an, China; 2 Shannxi Key Laboratory of Natural Products & Chemical Biology, College of Chemistry & Pharmacy, Northwest A & F University, Yangling, China; 3 Shaanxi Pharmaceutical Development Center, Xi’an, China

**Keywords:** Bufalin, Liposome, Tissue distribution, Antitumor efficacy, Acute toxicity

## Abstract

Bufalin, derived from *Venenum Bufonis*, exerts antitumor effects but has low bioavailability and adverse effects when administered as a single agent. The purpose of this study was to evaluate the physical and chemical properties, antitumor efficacy, general pharmacology, acute toxicity, and tissue distribution profile of bufalin-loaded PEGylated liposomes (BF/PEG-LP), which were prepared in a previous study. To evaluate the safety of the preparation, a red blood cell hemolysis test was performed, which indicated that the hemolysis rate of BF/PEG-LP was significantly lower than that of bufalin alone. Cell viability assay revealed that the blank liposomes were nontoxic. In an in vitro experiment, BF/PEG-LP dose-dependently induced the apoptosis of HepG2, HCT116, A549, and U251 cancer cells, with half-maximal inhibitory concentration (IC_50_) values of 21.40 ± 2.39, 21.00 ± 3.34, 43.39 ± 6.43, and 31.14 ± 2.58 ng/mL, respectively, at 24 h. Tumor xenograft experiments in nude mice showed that BF/PEG-LP significantly inhibited the growth of U251 cells. Pharmacological evaluation revealed that BF/PEG-LP impacted the general behavior, independent activities, and coordination of mice after a week of administration compared with those of mice in the control group. In an acute toxicity test, the median lethal concentration (LD_50_) of BF and BF/PEG-LP in mice was 0.156 and 3.03 mg/kg, respectively. Tissue distribution profiles showed that the BF concentration in brain tissue was 20% higher, whereas that in heart tissue was 30% lower when BF/PEG-LP was administered to mice compared with BF. Thus, BF/PEG-LP exhibited lower hemolysis and cytotoxicity and improved pharmacokinetic and antitumor properties compared with bufalin alone, indicating its potential for future pharmacological application, particularly for glioma treatment.

**Electronic supplementary material:**

The online version of this article (10.1186/s11671-019-3057-0) contains supplementary material, which is available to authorized users.

## Introduction

Bufalin (BF; 3β,14-dihydroxy-5β,20(22)-bufadienolide, 5β,20(22)-bufadienolide-3β, 14-diol), extracted from *Venenum Bufonis*, has anti-inflammatory, cardiotonic, antiviral, acesodyne, and antitumor activities [1, 2]. Our group also revealed that BF has therapeutic effects on hepatoma, melanoma, colonic carcinoma, glioma, and epithelial ovarian carcinoma, which could be attributed to its inhibition of cell proliferation and induction of cell apoptosis [3–7].

Lan et al. [8] previously established a glioma xenograft model in nude mice, to which BF was administered daily via intraperitoneal (i.p*.*) injection. In the BF-treated group, almost no binuclear kernels and mitosis were observed; in addition, nucleoli were smaller, and cell morphology was more regular than in the control group. Although BF has many potential pharmacological effects and the results obtained thus far are promising, BF as a single agent is still far from clinical application. Furthermore, BF treatment is associated with undesirable adverse and side effects such as immunoreaction, injury of normal tissues, and high toxicity [9].

Several drug delivery systems, such as lipid emulsions, nanoparticles, and liposomes, have been employed to promote the solubility, pharmacological activity, and bioavailability of BF [10, 11]. Moreover, the aggregation-induced emission (AIE) active functional polymeric self-assemblies have showed great potential for various biomedical applications including biological imaging and intracellular drug delivery [12, 13]. Among these drug delivery systems, liposomal preparations have several advantages for use as intravenous (i.v.) delivery systems. Liposomal preparations can not only improve the water solubility and reduce the side effects of the loaded agents [14], but also carry the agents across the blood-brain barrier (BBB) so as to treat the brain tumor more effectively and efficiently [15]. The BBB is composed of brain capillary endothelial cells and the tight junctions between them, star-shaped cells, glial cells, and the basement membrane, which enable only highly lipid-soluble drugs to pass and disperse in the brain tissue [16]. Nonetheless, liposome preparations have some shortcomings. Absorbance by plasma proteins and recognition by the mononuclear phagocytic system cause rapid clearance of the loaded drug from the blood. However, surface modification of liposomes with PEG could solve these problems [17].

In previous research, we prepared PEGylated liposomes loaded with bufalin (BF/PEG-LP) and characterized the same, including its cytotoxicity and pharmacokinetic profiles, in an attempt to overcome the abovementioned challenges, increase the efficacy of drug delivery, and decrease toxicity. To extend that research, we further evaluated the physical and chemical properties, antitumor efficacy, acute toxicity, and tissue distribution profiles of BF/PEG-LP.

## Materials and Methods

### Chemicals and Reagents

BF and cinobufagin (CBG) were purchased from BaoJi Chenguang Technology Development Co., Ltd. (BaoJi, China; 98% purity, identified by HPLC) and dissolved in DMSO for storage at − 20 °C as stock solutions. CBG was used as the internal standard (IS). Doxorubicin (DOX) was purchased from Beijing Solarbio Science & Technology Co., Ltd. (Beijing, China; 98% purity, identified by HPLC) and dissolved in DMSO for storage at − 20 °C as stock solutions. BF/PEG-LP were prepared in our laboratory (particle size, 155.0 ± 8.46 nm; zeta potential, – 18.5 ± 4.49 mV; entrapment efficiency, 76.31% ± 4.23%, detected by HPLC) [6]. HPLC-grade formic acid was purchased from Tedia Company Inc. (Fairfield, OH, USA). Cell counting kit-8 (CCK-8) was purchased from Dojindo Laboratories (Kumamoto, Japan). HPLC-grade ethyl acetate was purchased from Tianjin Kermel Chemical Reagents Development Centre (Tianjin, China). HPLC-grade acetonitrile was purchased from Merck (Darmstadt, Germany). All other reagents were of analytical grade.

### Animals

Male Sprague-Dawley rats, weighing approximately 230–250 g; Kunming mice, weighing 18–22 g; and 6-week-old nude Balb/c mice were supplied by the Experimental Animal Research Center, Fourth Military Medical University (Xi’an, China). The animals were kept separately in an individually ventilated cage (IVC) system and fed standard laboratory food and water ad libitum for 5 days. All animals were fasted (except for water) overnight before the experiments. Experimental procedures involving animals were reviewed and approved by the Institutional Animal Care and Use Committee of the Fourth Military Medical University (Approval 2017-0603-R).

### Preparation of Standard Samples

Calibration curves were prepared by spiking 100 μL of tissue homogenate with 20 μL each of the working solutions to produce samples containing 20, 50, 200, 500, and 2000 ng/mL of BF. The spiked standard homogenate samples were prepared in advance and evaluated with each analytical batch of unknown samples.

### Safety of the Liposomes

#### RBC Hemolysis Test

Eight milliliters of blood was collected from rats and subsequently heparinized and diluted with 10 mL 0.9% normal saline. The whole blood samples were then mixed with 100 μL of BF solution or 1 mL of BF/PEG-LP suspension. After 40 min of immersion in a water bath maintained at 37 °C, the samples were centrifuged at 750*g* for 5 min to remove the RBCs and fragments thereof. The supernatant was precipitated in 2 mL ethanol/hydrochloric acid solution (39:1; 99% ethanol (*v/v*):37% hydrochloric acid (*w/v*)). The supernatant was centrifuged at 750*g* for 5 min, after which the absorbance was measured at 398 nm by a UV spectrophotometer. A positive control consisting of distilled water and a negative control consisting of 0.9% saline were prepared and measured in the same manner.

The hemolysis rate (HR%) of the nanoparticle suspension was calculated as follows:

$$ \mathrm{HR}\%=\left({\mathrm{A}}_{\mathrm{sample}}-{\mathrm{A}}_{\mathrm{negative}}\right)/\left({\mathrm{A}}_{\mathrm{positive}}-{\mathrm{A}}_{\mathrm{negative}}\right)\times 100\% $$

where *A*_sample_ is the absorbance of the sample, *A*_negative_ is the absorbance of the negative control, and *A*_positive_ is the absorbance of the positive control.

#### Cytotoxicity of the blank liposomes

CCK-8 was used to detect the cytotoxicity of blank liposomes in human hepatocellular carcinoma HepG2 cells and human colon cancer HCT116 cells. Suspended HepG2 cells and HCT116 cells (1 × 10^4^ cells/well) were inoculated into a 96-well plate and cultured by RPMI-1640 culture medium until the logarithmic phase. Different concentrations of blank liposomes were added to the culture medium. Six replicate wells were prepared for each concentration. After incubation for 24 h, the medium was discarded, and 100 μL of CCK-8 working solution (CCK-8:RPMI-1640 culture medium, 10:100) was added. After 2 h of incubation, the absorbance was detected and quantified at 450 nm by a microplate reader (Bio-Rad 680, Hercules, CA, USA).

### Effect of Environmental pH on the Stability of Liposomes

BF/PEG-LP were prepared by hydration with phosphate-buffered saline (PBS) at pH 7.4 and pH 5.0, respectively. The liposomes were stored at 4 °C under dark conditions. The absorbance of samples at 296 nm was measured at 0, 5, 15, 30, 60, and 120 min.

### In Vitro Experiment

Cell proliferation was evaluated by CCK-8 proliferation assay. HepG2, HCT116, A549, and U251 cells were individually seeded at a density of 1 × 10^4^ cells/well in 96-well plates at 37 °C with 5% (*v/v*) CO_2_. After 24 h, different concentrations of BF/PEG-LP were added to the wells. After another 24 h of incubation, the cell medium was discarded, and 100 μL of CCK-8 working solution was added. After 2 h of incubation, the absorbance was detected and quantified at 450 nm by a microplate reader (Bio-Rad 680). The percentage of viable cells and IC_50_ values were estimated from a dose-response curve. Values of each sample and control were determined by the average of six replicate wells.

### Tumor Xenograft Experiment

U251 cells (1 × 10^7^) in 0.15 mL PBS were inoculated subcutaneously in nude mice and allowed to grow for 7 days to reach a tumor size of more than 50 mm^3^. Then, mice were randomly divided into two groups with six mice each. In the treatment group, 0.5, 1.0, or 2.0 mg/kg/day of BF or BF/PEG-LP was i.p. injected for 21 consecutive days. PBS containing 0.1% DMSO was injected in the vehicle control group, while 2 mg/kg of cisplatin (DPP) was injected in the positive control group.

The body weight of mice was measured every 2 days. All mice treated with BF and BF/PEG-LP were observed carefully for their skin and hair conditions, volume of food and water intake, fecal characteristics, and behavioral changes. At the end of the experiments, the mice were sacrificed, and the tumor xenografts were removed and weighed. The transplanted tumors were then fixed in 4% paraformaldehyde solution and embedded in paraffin. Five-micron-thick sections were prepared and stained with hematoxylin and eosin (H&E). The growth and necrosis of tumor cells and infiltration of surrounding tissues were observed under light microscopy.

### Pharmacological Evaluation

#### General Behavior and Independent Activities

Fifty Kunming mice were randomly divided into five groups (*n* = 10 per group) that were i.p. administered the following at a single dose: saline at the volume administered to the control group, 20 mg/kg pentobarbital sodium as the positive control group, and 1.0, 1.5, and 2.0 mg/kg as the low-, medium-, and high-dose BF/PEG-LP groups. The general behavior, posture, gait, salivation, myofibrillation, nystagmus, and secretions of mice were then observed. After 1 h of administration, the mice were placed in an open-field box. The number of mice moving in the box was counted for 10 min after the mice were allowed to adapt for 1 min.

#### Coordinated Exercise Tests

The mice were randomly divided into five groups (*n* = 10 per group, half male and half female) that were i.p. administered the following: saline at the volume administered to the blank control group, 2.5 mg/kg chlorpromazine hydrochloride as the positive control group; and 1.0, 1.5, and 2.0 mg/kg BF/PEG-LP as the low-, medium-, and high-dose BF/PEG-LP groups, respectively.

A smooth metal rod (diameter 1.27 cm, length 80 cm) was placed perpendicularly to the ground and fixed at the bottom to keep upright. After 1 week of treatment, the climbing test was performed for 30, 60, and 120 min, respectively. The mice were placed on the top of the metal rod and allowed to crawl down naturally. The coordination of mice as they climbed down the metal rod was observed and scored. The scoring criteria were as follows: 0 points, step-by-step descent; 1 point, sliding descent; 2 points, inability to descend; and 3 points, no reflex observed.

### Acute Toxicity

The median lethal concentration (LD_50_) of BF and BF/PEG-LP was calculated to evaluate acute toxicity. A total of 110 Kunming mice (half male and half female) were randomly divided into 11 groups (*n* = 10). Five groups received a single i.v. dose of BF at 1.0, 0.37, 0.14, 0.05, or 0.02 mg/kg, and five groups received a single i.v*.* dose of BF/PEG-LP at 4.0, 2.83, 2.0, 1.41, or 1.0 mg/kg. The injection volume was 0.2 mL. BF was first dissolved in normal saline with 1% DMSO; BF and BF/PEG-LP were then diluted with normal saline to obtain the desired concentration. The control group was administered normal saline containing 1% DMSO. After treatment, the general behavior of the mice was observed for 14 days, and the death rate was recorded. The pathological changes in the mice after i.v*.* administration were observed by optical microscopy (NIKON Eclipse ci, Tokyo, Japan).

#### Tissue Distribution Study Using HPLC

Sprague-Dawley rats were randomly assigned to two sections (four groups in each section, *n* = 6 per group). These two sections were i.p*.* administered 0.5 mg/kg BF and BF/PEG-LP, respectively. Samples of major tissues, including the heart, liver, spleen, lung, kidney, and brain, were collected at 5, 15, 45, and 90 min after administration. Tissue samples were quickly rinsed with saline solution (0.9%), removing the blood and contents as appropriate. After cleaning, filter paper was used to blot the samples. The samples were weighed and then homogenized in 0.9% saline solution (tissue sample to saline solution, 1:2, *w/w*). The obtained homogenates were stored at – 80 °C until analysis.

The target compounds were extracted, and the interference of endogenous protein was eliminated by a liquid-liquid preparation method [18]. We found that ethyl acetate improved the extraction recovery and minimized endogenous interference, resulting in enhanced sensitivity and selectivity of the detection of BF. Consequently, ethyl acetate was utilized as the optimal solvent for sample preparation. Twenty microliters of the IS working solution was added to a 100-μL aliquot of tissue homogenate. Then, the samples were vortex-mixed for 30 s and extracted with 2.5 mL ethyl acetate for 2 min. After centrifugation at 3500 g for 10 min, the upper organic layer was transferred to another tube and evaporated at 45 °C under a gentle stream of nitrogen. The residue was reconstituted in 100 μL mobile phase (acetonitrile: 0.1% formic acid/water solution, 65:35, *v/v*) though vortex-mixing for 5 min and centrifugation at 12000 g for 10 min. Then, a 10-μL aliquot of the supernatant was injected into the HPLC system (Waters 2995/2996, Waters Corporation, Milford, MA, USA). The optimized chromatograph conditions for detection of BF and IS were presented previously [6].

### Statistical Analysis

All results were expressed as mean ± SD (*n* = 3), and differences between formulations were compared by one-way analysis of variance (ANOVA), using GraphPad Prism 5 software. *P* < 0.05 denotes significance in all cases.

## Results and Discussion

### Physical and chemical properties of BF/PEG-LP

**Fig. 1:**
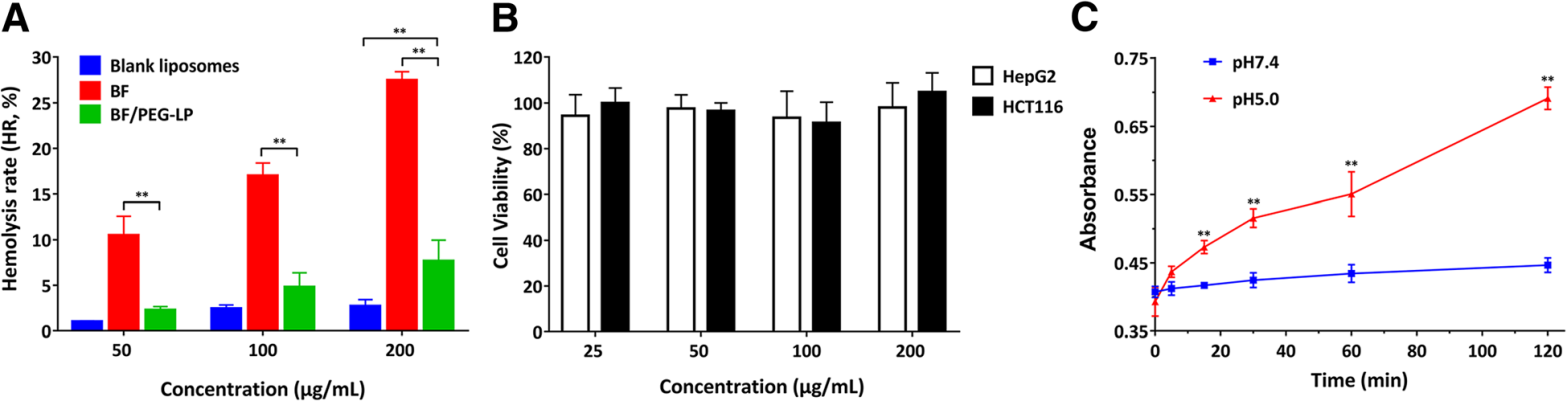
Physical and chemical properties of BF/PEG-LP. **a** Red blood cell hemolysis rate of blank liposomes, BF, and BF/PEG-LP. Data are presented as $\overline{x}$± s (*n* = 3). **P < 0.01 vs*.* BF/PEG-LP group at the same concentration. **b** Cytotoxicity of blank liposomes in HepG2 and HCT116 cells. Data are presented as $\overline{x}$ ± s (*n* = 6). **c** Stability of BF/PEG-LP in different pH conditions. Data are presented as $\overline{x}$ ± s (*n* = 6). **P < 0.01 vs*.* pH 7.4 group at same time point. Abbreviations: BF, bufalin; BF/PEG-LP, bufalin-loaded PEGylated liposomes

Transmission electron microscopy (TEM) revealed that BF/PEG-LP had a uniform spherical shape, as shown in Additional file 1: Figure S1A. The structure of BF/PEG-LP was identified by FT-IR. As shown in Additional file 1: Figure S1B, the characteristic peaks at 3495 cm^−1^ and 3436 cm^−1^ represented the N–H stretching vibration of the amide bond, while the peak at 1079 cm^−1^ represented the C–O–C stretching vibration of ether bond in BF/PEG-LP. To evaluate the safety of the formulations in vitro, the hemolysis rate of blank liposomes, BF, and BF/PEG-LP was determined by an RBC hemolysis test, and the cell viability of HepG2 and HCT116 cells treated with blank liposomes was measured by CCK-8 assay. The BF group exhibited obvious hemolysis; however, the hemolysis rate of the BF/PEG-LP group was significantly lower at the same concentration (*P* < 0.01). When the concentration of BF/PEG-LP was increased to 200 μg/mL, the hemolysis rate was obviously higher than that of blank liposomes (*P* < 0.01, Fig. 1a). The results of the CCK-8 assay showed that the blank liposomes were non-toxic on HepG2 and HCT116 cells (Fig. 1b).

The effect of environmental pH on the stability of BF/PEG-LP was evaluated by UV spectrophotometry. Figure 1c shows that the absorbance of the sample from the pH 5.0 group increased with storage time, indicating that BF/PEG-LP was more stable in PBS at pH 7.4 (*P* < 0.01).

### In Vitro Tumor Cell Apoptosis

**Fig. 2:**
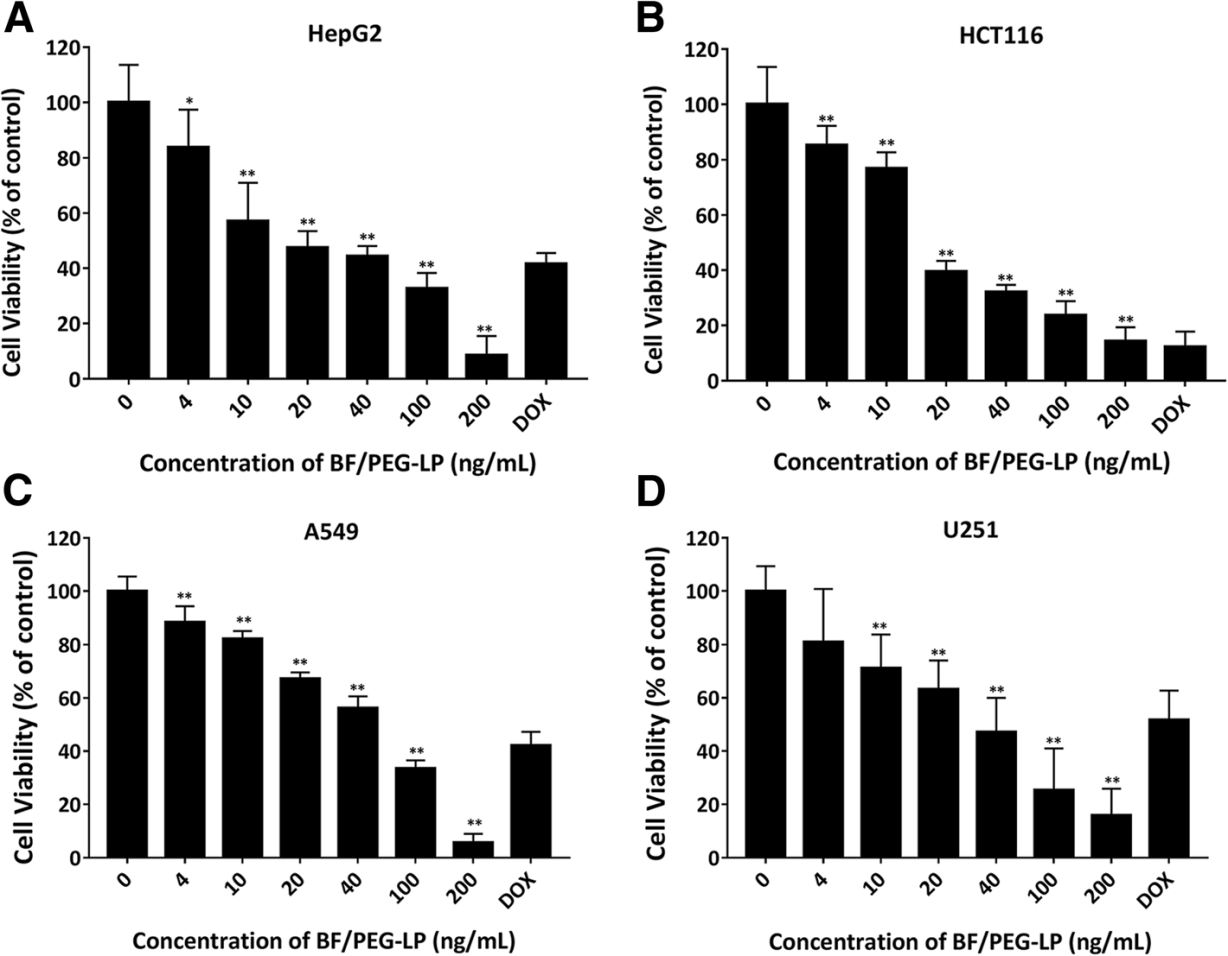
Cytotoxicity of BF/PEG-LP in HepG2 (**a**), HCT116 (**b**), A549 (**c**), and U251 (**d**) cells. Data are presented as$\overline{x}$ ± s (*n* = 6). *P < 0.05, **P < 0.01 vs*.* control group for each cell line. Abbreviations: BF/PEG-LP, bufalin-loaded PEGylated liposomes; DOX, doxorubicin

Apoptosis in HepG2, HCT116, A549, and U251 cells induced by BF/PEG-LP was determined by CCK-8 assay. The IC_50_ values of BF/PEG-LP for all tested cells are presented in Table 1. BF/PEG-LP significantly decreased the viability of all cell types in a dose-dependent manner (Fig. 2). The inhibitory effect of BF/PEG-LP was similar to that of DOX (positive control, 50 μg/mL) at concentrations exceeding 40 ng/mL. Moreover, based on the IC_50_ values, the sensitivity of cells to BF/PEG-LP was ranked as follows: HCT116 > HepG2 > U251 > A549.

**Table 1:** IC_50_ values of BF/PEG-LP for cancer cell lines

Cell lines	IC_50_ (ng/mL)
HepG2	21.40 ± 2.39
HCT116	21.00 ± 3.34
A549	43.39 ± 6.43
U251	31.14 ± 2.58

Values are $$ \overline{x} $$± s (*n* = 3)

*IC*_*50*_, half-maximal inhibitory concentration

### General Pharmacological Evaluation

**Fig. 3:**
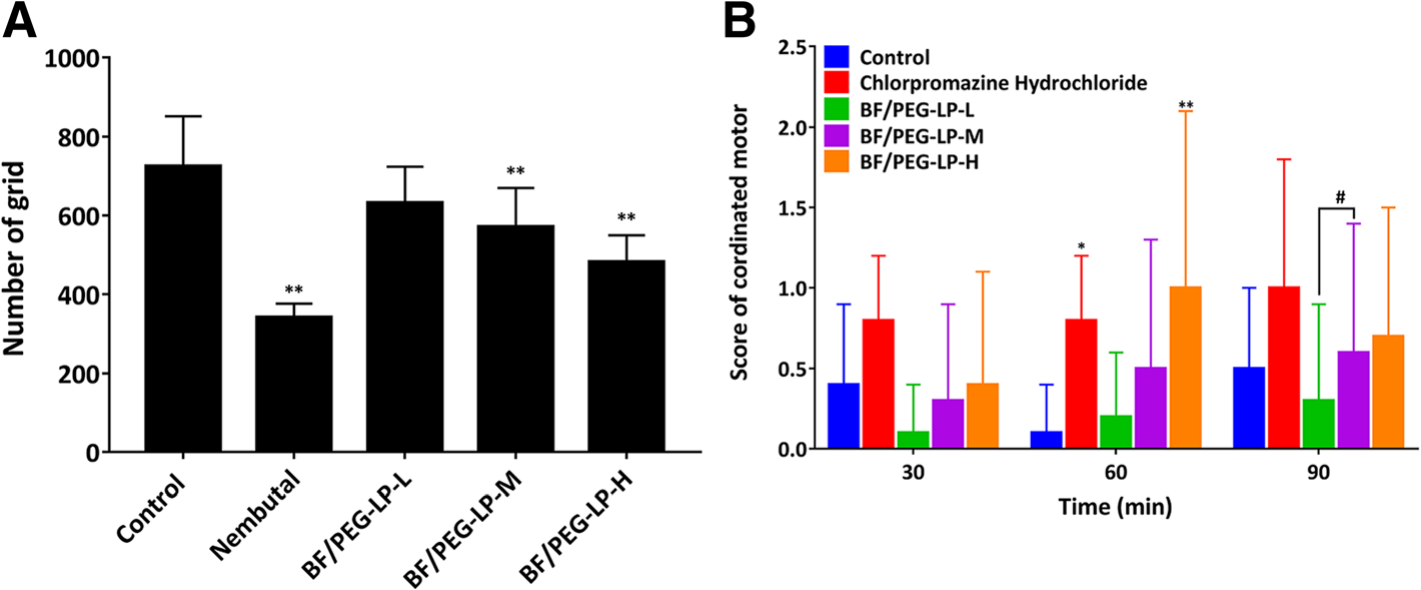
Pharmacological effect of BF/PEG-LP on locomotor activity (**a**) and coordinated exercise (**b**) in mice. Data are presented as $\overline{x}$ ± s (*n* = 10). *P < 0.05, *^*^P < 0.01 vs*.* control group at each time point; ^*#*^*P* < 0.05 vs. low-dose BF/PEG-LP group. Abbreviations: BF/PEG-LP, bufalin-loaded PEGylated liposomes

After administration of BF/PEG-LP, the general behavior of mice was different from that of the blank group. In particular, the mice in the high-dose BF/PEG-LP group exhibited tremors and salivation. After administration, the mice remained in the open-field box for 10 min, and the number of active grids was recorded. In the positive control (pentobarbital) group, the number of active grids was only 342 ± 35, which was significantly lower than that in the control group (725 ± 127, *P* < 0.05). As shown in Fig. 3a, the numbers of active grids in the medium- and high-dose BF/PEG-LP group were significantly different from the control group. The rod-climbing test showed that BF/PEG-LP had a certain promoting effect on the coordinated movement of mice after a week of administration compared with that of the control group (*P* < 0.01), while chlorpromazine had a significant promoting effect on the positive control group (*P* < 0.05), as shown in Fig. 3b.

### Tumor Xenograft Experiment

**Fig. 4:**
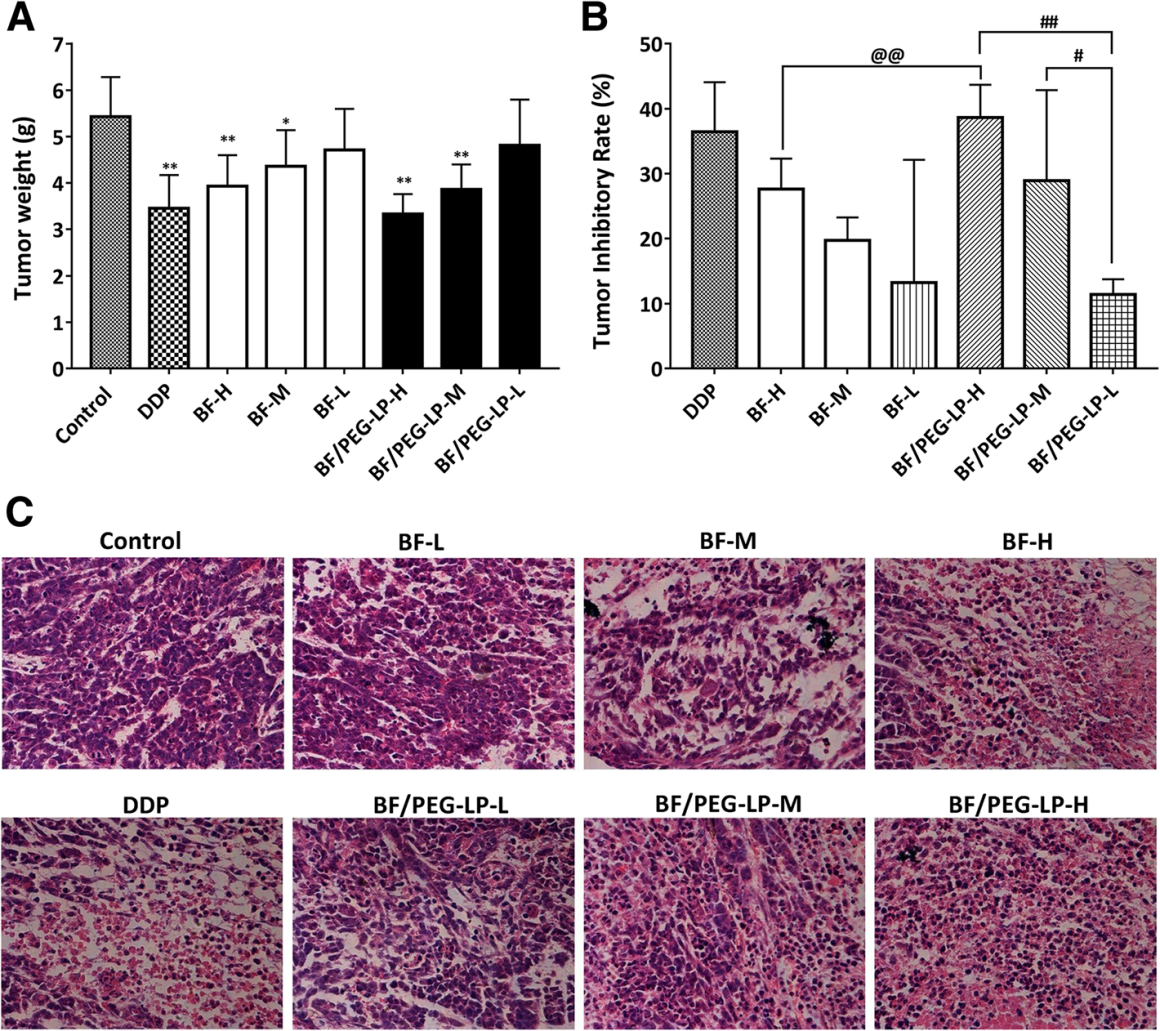
Inhibitory effect of BF and BF/PEG-LP on the growth of glioma xenografts in nude mice. Comparison of the tumor weight (**a**) and tumor inhibition rate (**b**) in each group. Data are presented as $\overline{x}$ ± s (*n* = 10). *P < 0.05, **P < 0.01 *vs.* control group; ^*#*^*P* < 0.05, ^*##*^*P* < 0.01 vs*.* high-dose BF/PEG-LP group. **c** H&E staining of tumor tissues from tumor-bearing mice. Abbreviations: BF, bufalin; BF/PEG-LP, bufalin-loaded PEGylated liposomes; DPP, cisplatin; H&E, hematoxylin and eosin

With prolonged administration, mice in the positive control, BF, and BF/PEG-LP groups showed mental retardation, reduced food intake, slow reaction, and dull fur. Mouse death occurred in both the BF and BF/PEG-LP groups. The antitumor rate of the positive control group was 36.5%; that of the high-dose BF group was 27.6%; and that of the low-, medium-, and high-dose BF/PEG-LP groups was 11.4%, 28.9%, and 38.7%, respectively. Except for the low-dose BF group, there was a significant difference between the antitumor rates of the drug-treated groups and the negative control group (*P* < 0.05), as shown in Fig. 4a. Compared with that of the low-dose BF group, the tumor inhibition rate of the high-dose BF/PEG-LP group was significantly higher (*P* < 0.01), as shown in Fig. 4b.

The xenografts in the tumor-bearing mice were nodular in shape. H&E staining showed that in the control group, the nuclei of the tumor cells were large, the cytoplasm was small, and the growth of cells was vigorous; in the high-dose BF/PEG-LP group, a large area of necrotic tumor tissue was observed, and the degree of necrosis was dose-dependent, as shown in Fig. 4c.

### Acute Toxicity

The acute toxicity of a single i.v*.* dose of the formulations in Kunming mice was determined. The LD_50_ values of BF and BF/PEG-LP were 0.156 and 3.03 mg/kg, respectively. Compared with that of BF, the acute toxicity of BF/PEG-LP was significantly lower (*P* < 0.01); this effect was attributed to the liposomal formulation, which controlled BF release into the blood.

**Fig. 5:**
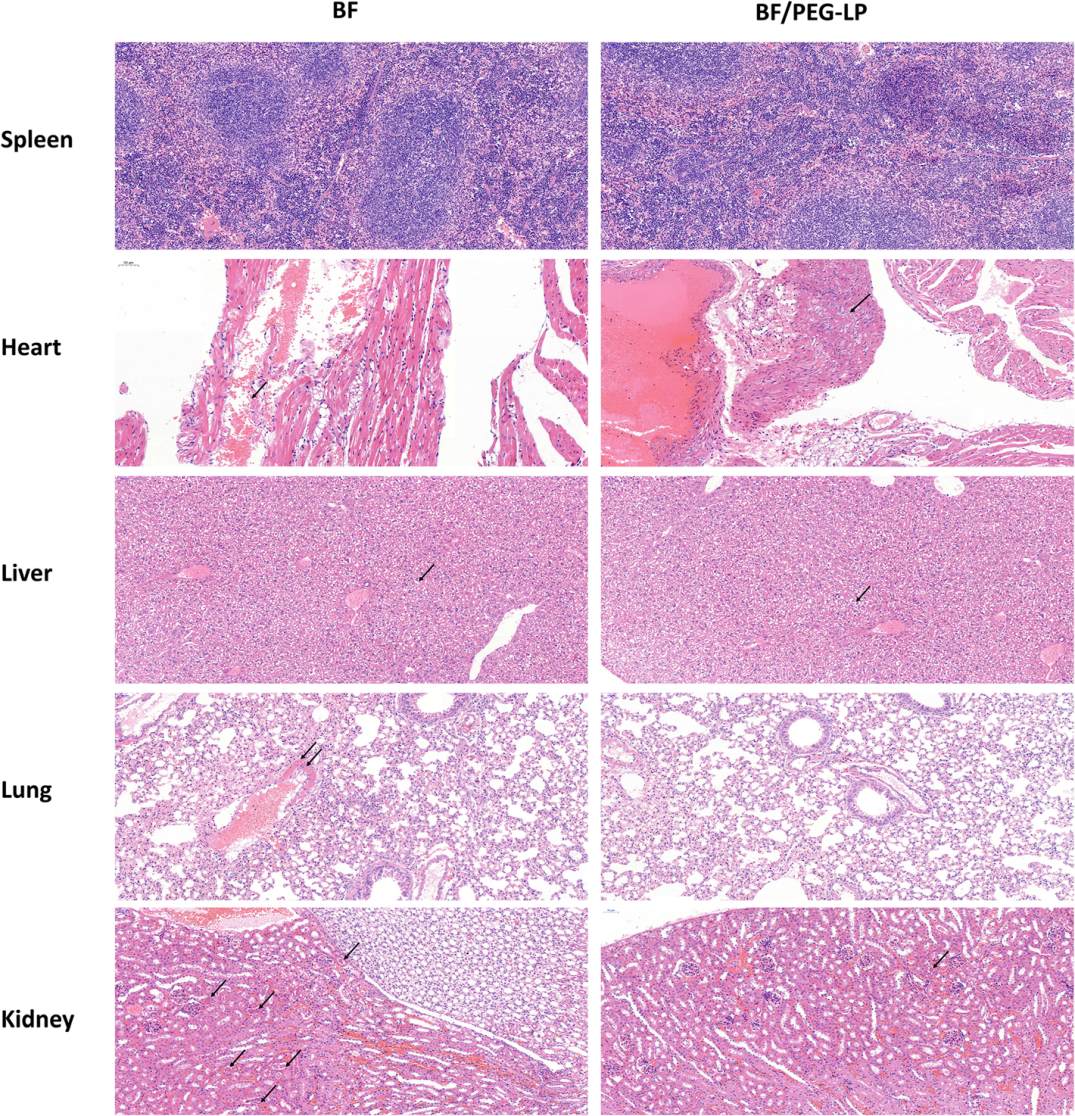
Pathological changes observed in dead mice after intravenous administration of 1.0 mg/kg BF and 4.0 mg/kg BF/PEG-LP. Tissue sections were stained with H&E and observed by optical microscopy. Notes: Optical microscope: NIKON Eclipse ci; Imaging system: NIKON Digital Sight DS-FI2 (Tokyo, Japan); Magnification: ×200. Abbreviations: BF, bufalin; BF/PEG-LP, bufalin-loaded PEGylated liposomes; H&E, hematoxylin and eosin

The pathological changes in the mice after i.v*.* administration were observed by optical microscopy (Fig. 5). Pathological changes were observed in several organs, such as the heart, liver, and kidney. Heart myocardial fiber dissolution and fracture with bleeding were obvious. Necrosis was observed in hepatocytes and pulmonary endotheliocytes.

### Tissue Distribution Study

Comparison of the chromatograms of the blank tissue homogenate and spiked homogenate, which indicated that there was no significant interference at the retention times of BF and CBG (IS), was used to assay the selectivity of the method, as shown in Additional file 1: Figure S2. Calibration curves were constructed from the peak-area ratios of BF to IS (Y) versus tissue homogenate concentrations ranging from 20 to 2000 ng/mL, with a correlation coefficient *R*^2^ > 0.9977 (Table 2). The precision, accuracy and recovery experiments of BF in biological samples were shown in Additional file 1: Table S1, while the stability experiment results were shown in Additional file 1: Table S2.

**Table 2:** Typical equations for the calibration curves

Biosamples	Standard curve	Linear range (ng/mL)	Correlation coefficient
Heart	*Y* = 0.0044*x* + 0.006	20–2000	*R*^2^ = 0.9994
Liver	*Y* = 0.0034*x* + 0.0166	20–2000	*R*^2^ = 0.9997
Spleen	*Y* = 0.0041*x* + 0.015	20–2000	*R*^2^ = 0.9996
Lung	*Y* = 0.0036*x* + 0.0226	20–2000	*R*^2^ = 0.9994
Kidney	*Y* = 0.0036*x* + 0.0032	20–2000	*R*^2^ = 0.9977
Brain	*Y* = 0.0026*x* + 0.100	20–2000	*R*^2^ = 0.9997

**Fig. 6:**
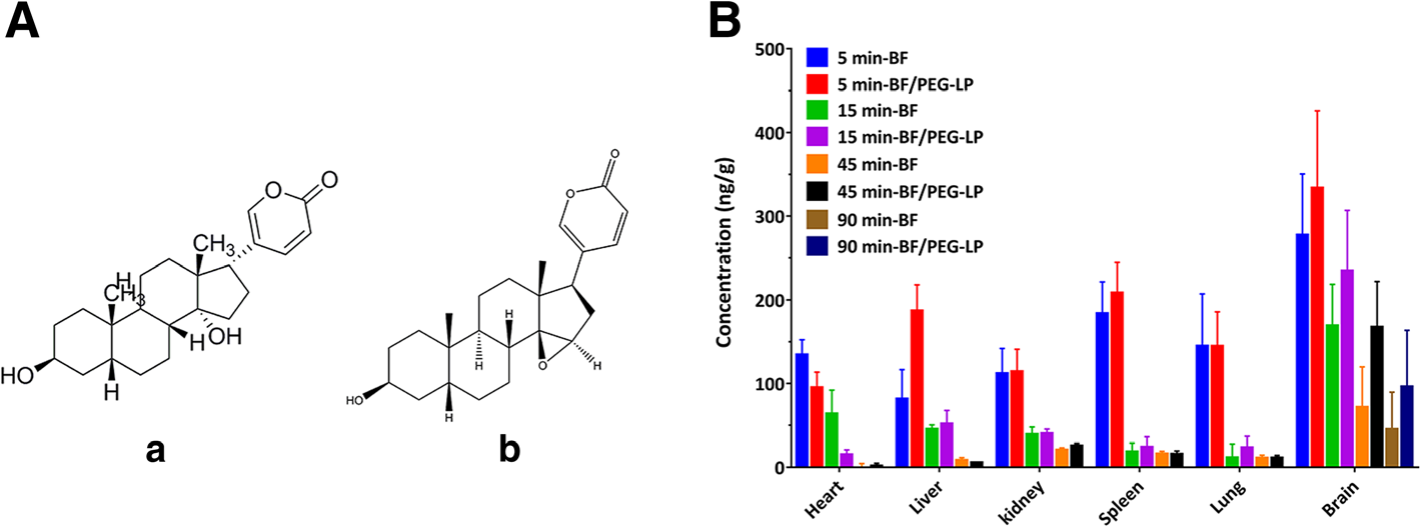
Tissue distribution of BF in rat organs. **A** Molecular formulas of bufalin (**a**) and cinobufagin (**b**, IS). **B** Tissue distribution profiles of BF in rats after a single intravenous dose of BF/PEG-LP or BF at 0.5 mg/kg. Data are presented as $\overline{x}$ ± s (*n* = 6). Abbreviations: BF, bufalin; BF/PEG-LP, bufalin-loaded PEGylated liposomes; IS, internal standard

The raw data of the tissue distribution experiments are shown in Additional file 1: Table S3. After i.v*.* injection of BF/PEG-LP, the tissue distribution thereof is influenced by several variables, such as composition, particle size, and zeta potential [19]. The concentration of BF in various tissues, including the heart, liver, spleen, lung, kidney, and brain, were quantified at 5, 15, 45, and 90 min, as shown in Fig. 6. At 45 min after i.v*.* administration, the concentration of BF in both groups was very low in some tissues, such as the heart, liver, spleen, lung, and kidney, indicating that the distribution and elimination of BF occurred quickly. The concentration of BF in the brain, though not other tissues, could still be detected at 90 min after i.v*.* administration in both groups, indicating that the distribution and elimination of BF were relatively slower in the brain than other tissues.

Meanwhile, 1.20-fold higher BF (333 ± 92.5 ng/g at 5 min) was detected in the brain tissues of the BF/PEG-LP group compared with that in the BF group. In addition, a higher concentration of BF was found in the liver, spleen, and brain tissues in the BF/PEG-LP group. Significant accumulation of BF was detected in the liver, with concentrations of 187 ± 31.0 ng/g in the BF/PEG-LP group and 163 ± 35.0 ng/g in the BF group (*P* < 0.01). A similar phenomenon, which is mainly caused by a dramatic increase in BF in the blood before metabolism and excretion, was observed in previous studies [20, 21].

According to the results of the tissue distribution experiments, BF itself could traverse the BBB, possibly owing to its high lipid solubility, low molecular weight (386.52 g/mol), and chemical structure, which is similar to that of cholesterol, with which it shares the same mother nuclear structure. However, the clinical application of BF is limited because of its high lipid solubility and poor water solubility. The bioavailability of BF is low if not administered in an alternative form. In a previous study [6], we showed that the BF/PEG-LP formulation could improve the water solubility, extend the blood circulation duration, prolong the half-life, and expand the scope of application of BF, including for i.v. chemotherapy. In the current study, BF/PEG-LP administration resulted in an increased concentration of BF in the brain, which was sustained for a longer duration (90 min). Moreover, there was a significant difference in BF concentration between the BF and BF/PEG-LP groups at 45 min (167.59 ± 54.52 *vs.* 71.52 ± 48.35 ng/g, *P* < 0.01).

In a previous study, BF was demonstrated to exert a digitalis-like effect on the heart, increasing the heart rate and myocardial contractive power in rat [22]. Therefore, the cardiotoxicity of BF at large doses may be caused by its accumulation in the heart. The biodistribution experiment in this study showed that the accumulation of BF in the heart was lower in the BF/PEG-LP group than in the BF group (95.0 ± 18.5 *vs*. 134 ± 17.8 ng/g at 5 min, *P* < 0.01; 15.32 ± 5.56 *vs.* 63.79 ± 28.21 ng/g at 15 min, *P* < 0.01). Whether cardiac toxicity can be attenuated with a lower dose of BF requires further verification. Nevertheless, the antitumor effect of BF was sufficiently strong in this study (the IC_50_ values for glioma cells were nanomolar). For in vivo application, it is critical to decrease the toxicity (especially heart toxicity) and increase the water solubility of BF, which was our focus in this study.

In general, PEGylated liposomes are considered to have no or low immunogenicity. However, according to recent studies, when PEGylated liposomes were repeatedly injected into the same animal, an immune response was triggered [23]. In fact, a second injection of PEGylated liposomes resulted in reduced blood circulation time and increased hepatic and splenic accumulation, which is known as the accelerated blood clearance (ABC) phenomenon. Such immunogenicity of PEGylated liposomes presents major challenges in the research of liposomal formulations and their clinical application. However, liposomal formulations that do not display the ABC phenomenon have been reported, such as a long-circulating DOX-loaded liposomal formulation (Doxil, Sequus pharmaceuticals, Inc., San Francisco, CA, USA), which has been commercialized. DOX was released from the liposomes and entered the spleen, damaging spleen cells and thereby reducing the production of anti-PEG IgM, which could selectively bind with PEG on the surface of the liposomes administered at the second injection. Whether BF/PEG-LP exhibits the ABC phenomenon remains unknown and requires further study. If so, mitigating the ABC phenomenon will require in-depth investigation.

## Conclusion

In the present study, we evaluated hemolysis induced by BF/PEG-LP and the cytotoxicity of blank liposomes to verify the safety thereof. Moreover, the effect of environmental pH on the release of BF from liposomes was investigated to assess the stability of BF/PEG-LP. BF encapsulation in a liposomal suspension might affect its antitumor activity in vitro and in vivo and general behavior, acute toxicity, and tissue distribution in vivo. Nonetheless, we believe that the development of the liposomal formulation described in this study has laid a foundation for the future clinical application of BF. Intensive investigation of the relevance of the ABC phenomenon to BF/PEG-LP is warranted in the future.

## Additional file

Additional file 1: **Figure S1.** Characterization of PEGylated liposomes containing bufalin. **Figure S2.** Representative chromatograms of BF and CBG (IS) in rat tissue homogenate samples. **Table S1.** The precision, accuracy and recovery experiments of bufalin in biological samples (*n* = 5). **Table S2.** The stability experiments of bufalin in biological samples (*n* = 5). **Table S3.** The tissue distribution of BF in rat organs. (DOCX 822 kb)

## Data Availability

All data generated or analyzed during this study are included in this published article and its supplementary information files.

## Abbreviations

ABC: Accelerated blood clearance
AIE: The aggregation-induced emission
BBB: Blood-brain barrier
BF: Bufalin
BF/PEG-LP: Bufalin-loaded PEGylated liposomes
CBG: Cinobufagin
CCK-8: Cell counting kit-8
IC_50_: Half-maximal inhibitory concentration
i.p.: Intraperitoneally
IS: Internal standard
i.v*.*: Intravenously
LD_50_: Median lethal concentration
PBS: Phosphate-buffered saline
RBC: Red blood cell
