# Supplementary material for: Bufalin-Loaded PEGylated Liposomes: Antitumor Efficacy, Acute Toxicity, and Tissue Distribution
Source: Nanoscale Res Lett. 2019 Jul 5;14:223. doi: 10.1186/s11671-019-3057-0 (PMC6611856; doi:10.1186/s11671-019-3057-0)
Supplement: Supplementary file 1 — Figure S1. Characterization of PEGylated liposomes containing bufalin. Figure S2. Representative chromatograms of BF and CBG (IS) in rat tissue homogenate samples. Table S1. The precision, accuracy and recovery experiments of bufalin in biological samples (n = 5). Table S2. The stability experiments of bufalin in biological samples (n = 5). Table S3. The tissue distribution of BF in rat organs. (DOCX 822 kb) [file 11671_2019_3057_MOESM1_ESM.docx]

**Additional file 1**

**
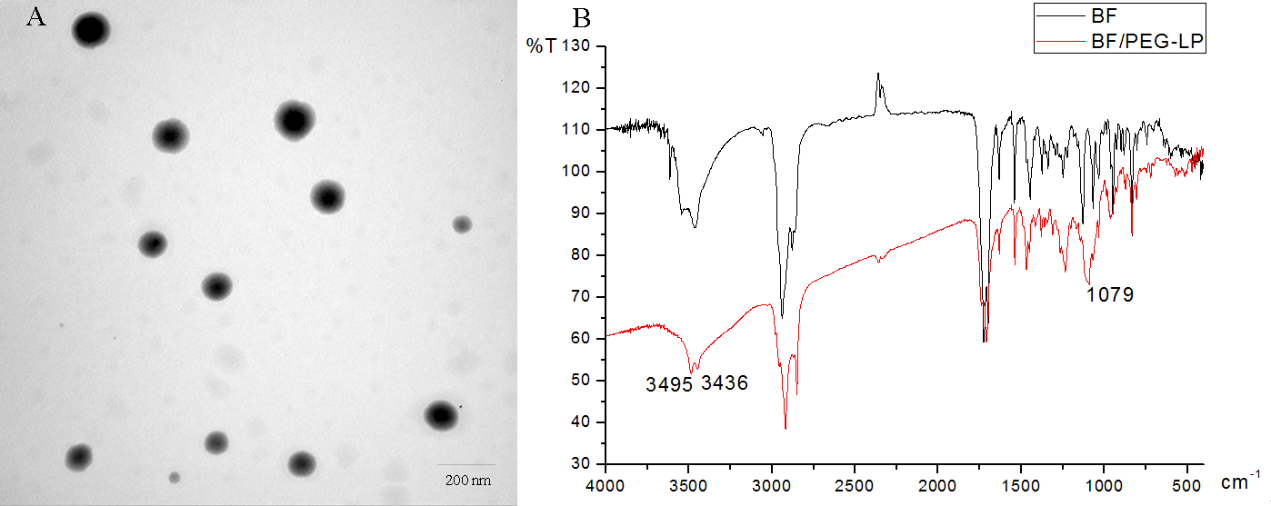
**

Figure S1 characterization of PEGylated liposomes containing bufalin**.** (A) TEM images of BF/PEG-LP, with a uniform spherical shape; (B) The characteristic peaks of BF/PEG-LP identified by FT-IR.

Abbreviations: BF/PEG-LP, bufalin-loaded PEGylated liposomes; TEM, transmission electron microscope; FT-IR, Fourier Transform Infrared Spectrometer.


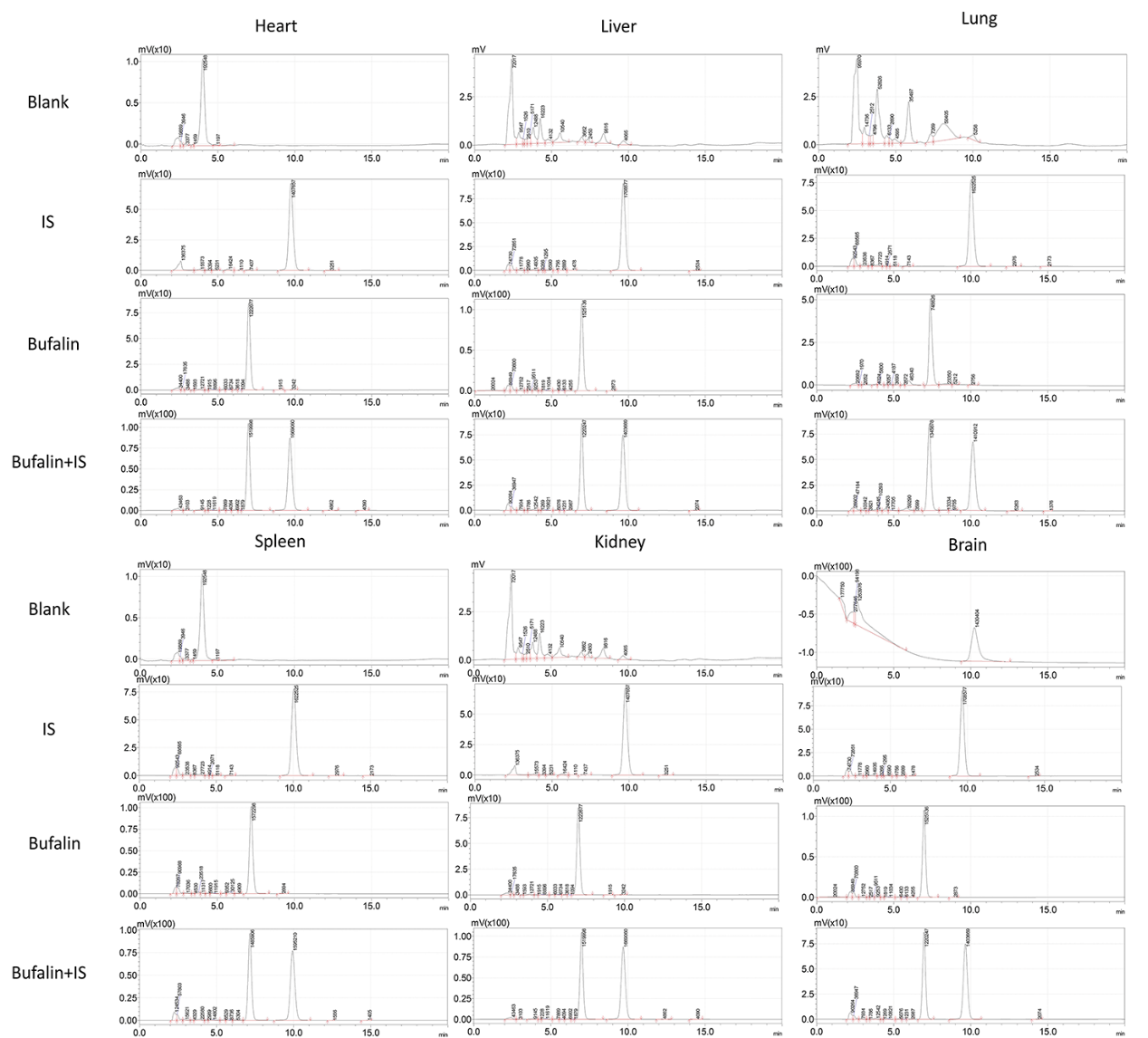
Figure S2 Representative chromatograms of BF and CBG (IS) in rat tissue homogenate samples.

Abbreviations: BF, bufalin; CBG, cinobufagin; IS, internal standard

Table S1 The precision, accuracy and recovery experiments of bufalin in biological samples (n=5)

| Biological samples | Added conc. (ng/mL) | Intra-day | | | Inter-day | | | Recovery | |
| --- | --- | --- | --- | --- | --- | --- | --- | --- | --- |
|  |  | Mean conc. (ng/mL) | Precision  (%, RSD) | Accuracy  (%, RE) | Mean conc. (ng/mL) | Precision  (%, RSD) | Accuracy  (%, RE) | Mean  (%) | RSD  (%) |
| Heart | 40.0 | 38.9 | 5.9 | -2.8 | 43.5 | 6.7 | 8.75 | 103.8 | 6.6 |
|  | 200.0 | 205.4 | 6.6 | 2.7 | 211.4 | 11.3 | 5.7 | 96.7 | 12.6 |
|  | 1000.0 | 994.1 | 5.2 | -0.6 | 1033.4 | 8.4 | 3.4 | 108.4 | 10.4 |
| Liver | 40.0 | 42.6 | 8.9 | 6.5 | 45.7 | 12.4 | 14.3 | 94.3 | 7.8 |
|  | 200.0 | 197.4 | 5.4 | -1.3 | 229.8 | 9.8 | 14.9 | 98.7 | 2.5 |
|  | 1000.0 | 1004.5 | 6.8 | 0.5 | 1045.7 | 12.4 | 4.6 | 110.8 | 4.6 |
| Spleen | 40.0 | 41.0 | 7.0 | 2.5 | 38.1 | 11.3 | -4.8 | 91.0 | 12.5 |
|  | 200.0 | 187.7 | 4.5 | 6.2 | 205.5 | 10.2 | 2.8 | 112.5 | 11.4 |
|  | 1000.0 | 984.3 | 3.9 | -1.6 | 1081.4 | 11.6 | 8.2 | 108.6 | 10.6 |
| Lung | 40.0 | 37.4 | 6.0 | -6.5 | 43.9 | 7.9 | 9.8 | 103.4 | 6.1 |
|  | 200.0 | 208.9 | 4.3 | 4.5 | 213.0 | 12.5 | 6.5 | 113.5 | 13.0 |
|  | 1000.0 | 1014.6 | 6.5 | 1.5 | 1054.7 | 10.1 | 5.5 | 102.6 | 6.7 |
| Kidney | 40.0 | 44.5 | 6.9 | 11.25 | 45.9 | 10.5 | 14.8 | 97.4 | 4.8 |
|  | 200.0 | 182.4 | 3.4 | 8.8 | 204.1 | 12.3 | 2.1 | 95.3 | 9.8 |
|  | 1000.0 | 1023.4 | 6.0 | 2.3 | 1031.0 | 14.4 | 3.1 | 97.1 | 13.1 |
| Brain | 40.0 | 43.8 | 7.7 | 7.5 | 43.7 | 8.3 | 9.25 | 110.0 | 10.5 |
|  | 200.0 | 214.7 | 5.6 | 7.4 | 203.3 | 10.5 | 1.7 | 106.4 | 8.7 |
|  | 1000.0 | 977.1 | 6.4 | -2.3 | 1051.0 | 12.6 | 5.1 | 108.7 | 10.0 |

Table S2 The stability experiments of bufalin in biological samples (n=5)

| Biological samples | Added conc. (ng/mL) | Room temperature for 1 day | | -80℃ for 1 day | | Frozen thaw 3 times | |
| --- | --- | --- | --- | --- | --- | --- | --- |
|  |  | Mean (%) | RSD  (%) | Mean (%) | RSD  (%) | Mean (%) | RSD  (%) |
| Heart | 40.0 | 42.3 | 6.3 | 38.7 | 8.6 | 41.3 | 12.6 |
|  | 200.0 | 201.8 | 8.4 | 208.5 | 6.4 | 197.6 | 4.4 |
|  | 1000.0 | 997.4 | 6.2 | 1036.1 | 13.3 | 1033.6 | 6.8 |
| Liver | 40.0 | 40.8 | 7.4 | 40.6 | 6.5 | 42.5 | 8.9 |
|  | 200.0 | 198.5 | 3.6 | 184.3 | 8.9 | 213.4 | 9.6 |
|  | 1000.0 | 1016.7 | 5.9 | 976.4 | 14.0 | 1054.0 | 10.4 |
| Spleen | 40.0 | 43.6 | 3.3 | 41.5 | 5.2 | 40.6 | 4.5 |
|  | 200.0 | 206.8 | 8.0 | 211.2 | 6.1 | 228.4 | 8.0 |
|  | 1000.0 | 1034.5 | 7.8 | 1033.7 | 7.7 | 1006.5 | 1.9 |
| Lung | 40.0 | 43.1 | 4.5 | 40.1 | 6.4 | 39.5 | 6.6 |
|  | 200.0 | 211.0 | 4.6 | 186.5 | 5.5 | 202.7 | 11.3 |
|  | 1000.0 | 987.4 | 5.4 | 1025.8 | 6.3 | 948.3 | 8.0 |
| Kidney | 40.0 | 37.5 | 6.5 | 41.3 | 8.0 | 44.6 | 5.8 |
|  | 200.0 | 187.3 | 8.2 | 207.4 | 10.3 | 214.5 | 9.3 |
|  | 1000.0 | 1035.4 | 6.8 | 993.0 | 6.7 | 938.6 | 6.8 |
| Brain | 40.0 | 44.1 | 3.5 | 38.7 | 3.6 | 43.7 | 9.1 |
|  | 200.0 | 213.9 | 4.4 | 205.8 | 5.1 | 201.4 | 12.4 |
|  | 1000.0 | 1010.0 | 6.1 | 1089.4 | 10.3 | 1002.6 | 13.6 |

Table S3 The tissue distribution of BF in rat organs

|  | BF/PEG-LP | | | BF | | | |
| --- | --- | --- | --- | --- | --- | --- | --- |
| Heart-5min | 95.02 | 18.54 | 6 | | 134.5 | 17.84 | 6 |
| Heart-15min | 15.32 | 5.56 | 6 | | 63.79 | 28.21 | 6 |
| Heart-30min | -0.83 | 3.03 | 6 | | 0.01 | 4.48 | 6 |
| Liver-5min | 187.15 | 31.02 | 6 | | 81.53 | 35.01 | 6 |
| Liver-15min | 51.79 | 15.95 | 6 | | 45.47 | 4.94 | 6 |
| Liver-30min | -9.77 | 0.01 | 6 | | -8.52 | 3.04 | 6 |
| kidney-5min | 114.33 | 26.65 | 6 | | 112.08 | 29.82 | 6 |
| kidney-15min | 20.26 | 5.18 | 6 | | 19.34 | 8.61 | 6 |
| kidney-30min | -0.64 | 2.78 | 6 | | -0.8 | 2.39 | 6 |
| Spleen-5min | 208.7 | 35.91 | 6 | | 183.98 | 37.7 | 6 |
| Spleen-15min | 24.05 | 12.24 | 6 | | 18.64 | 10.29 | 6 |
| Spleen-30min | -5.83 | 3.62 | 6 | | -6.08 | 3.02 | 6 |
| Lung-5min | 144.83 | 40.99 | 6 | | 144.96 | 62.31 | 6 |
| Lung-15min | 23.56 | 13.45 | 6 | | 11.49 | 16.38 | 6 |
| Lung-30min | -11.41 | 2.8 | 6 | | -11.14 | 3.46 | 6 |
| Brain-5min | 333.73 | 92.47 | 6 | | 277.45 | 73.08 | 6 |
| Brain-15min | 235 | 71.86 | 6 | | 169.36 | 49.24 | 6 |
| Brain-30min | 167.59 | 54.52 | 6 | | 71.52 | 48.35 | 6 |
| Brain-90min | 95.98 | 67.61 | 6 | | 45.3 | 44.19 | 6 |
